# Genetic Predisposition to Low-Density Lipoprotein Cholesterol May Increase Risks of Both Individual and Familial Alzheimer's Disease

**DOI:** 10.3389/fmed.2021.798334

**Published:** 2022-01-11

**Authors:** Jiang-Shan Tan, Meng-Jin Hu, Yan-Min Yang, Yue-Jin Yang

**Affiliations:** State Key Laboratory of Cardiovascular Disease, National Center for Cardiovascular Diseases, Fuwai Hospital, Chinese Academy of Medical Sciences and Peking Union Medical College, Beijing, China

**Keywords:** low-density lipoprotein cholesterol, Alzheimer's disease, family history, mendelian randomization, familial Alzheimer's disease

## Abstract

**Background:** Previous observational studies provided conflicting results on the association between low-density lipoprotein cholesterol (LDL-C) level and the risk of Alzheimer's disease (AD).

**Objective:** We used two-sample Mendelian randomization (MR) study to explore the causal associations between LDL-C level and the risks of individual, paternal, maternal, and family history of AD.

**Methods:** Summary-level genetic data for LDL-C were acquired from results of the UK Biobank GWAS. Corresponding data for paternal, maternal, and family history of AD were obtained from the NHGRI-EBI Catalog of human genome-wide association studies. Data for individual AD were obtained from the MR-Base platform. A two-sample MR study was performed to explore the causal association between LDL-C level and the risks of individual, paternal, maternal, and family history of AD.

**Results:** Genetically predicted LDL-C was positively associated with individual [Odds ratio (OR) = 1.509, 95% confidence interval (CI) = 1.140–1.999; *P* = 4.0 × 10^−3^], paternal [OR = 1.109, 95% CI = 1.053–1.168; *P* = 9.5 × 10^−5^], maternal [OR = 1.132, 95% CI = 1.070–1.199; *P* = 2.0 × 10^−5^], and family history of AD [OR = 1.124, 95% CI = 1.070–1.181; *P* = 3.7 × 10^−6^] in inverse variance weighted analysis. After performing weighted median and MR-Egger analysis, consistent results were observed. There was no horizontal pleiotropy in the two-sample MR analysis.

**Conclusions:** High level of LDL-C may increase the risks of both individual and familial AD. Decreasing the LDL-C to a reasonable level may help to reduce the related risk.

## Introduction

Alzheimer's disease (AD), the most prevalent type of neurodegenerative disorder, is characterized by a progressive and irreversible decline in memory, thinking, and cognitive skills. It is reported that approximately 50 million people are suffering from dementia worldwide, in which AD accounts for 50–70% and increases the risk of morbidity (1). However, trials for drugs designed to modify AD have yielded disappointing results. There are still no effective therapies that can either reliably slow down the onset of AD or alleviate, delay or halt the clinical progression of the disease. At present, the primary prevention of AD focus on the modification of vascular risk factors, including dyslipidemia, blood pressure, fasting glucose, or weight (2, 3). As lipid fractions represent easily modifiable potential targets for prevention, exploring their relationship with AD risk is of great significance. Low-density lipoprotein cholesterol (LDL-C), a type of lipoprotein particle that carries cholesterol into cells of peripheral tissue, is well-established in the development of atherosclerotic cardiovascular disease (4). Lowering LDL-C level has been demonstrated to reduce the risks of myocardial infarction (MI) and stroke (5, 6). However, there remains no consensus on the association between elevated LDL-C level and the risk of AD. Some studies reported that patients with AD exhibited a higher level of LDL-C than normal controls (7, 8). In contrast, other studies reported no significant difference in LDL-C level between AD patients and healthy controls (9, 10). However, the correlation between LDL-C level and AD risk cannot be reliably interpreted in the abovementioned observational studies because these findings may be influenced by potential confounding factors, which is a significant limitation of observational studies. Therefore, the true association between LDL-C level and AD risk may be obscured. Moreover, the increased risk of AD at a high LDL-C level may also be due to reverse causation, as preclinical AD may change lifestyle and thereafter LDL-C level (11).

The Mendelian randomization (MR) is an epidemiological approach that aims to circumvent confounding and reverse causation by using genetic instrumental variables as proxies for environmental exposures in populations. Because the genotypes are randomly allocated from parents to offspring, independent of confounding factors that influence the risk factors and unaffected by reverse causation, MR is akin to conducting a genetic randomized control trial (12). Using two-sample MR analysis, we are determined to dissect the causal association between LDL-C level and AD risk.

## Materials and Methods

### Overall Study Design

**Figure 1:**
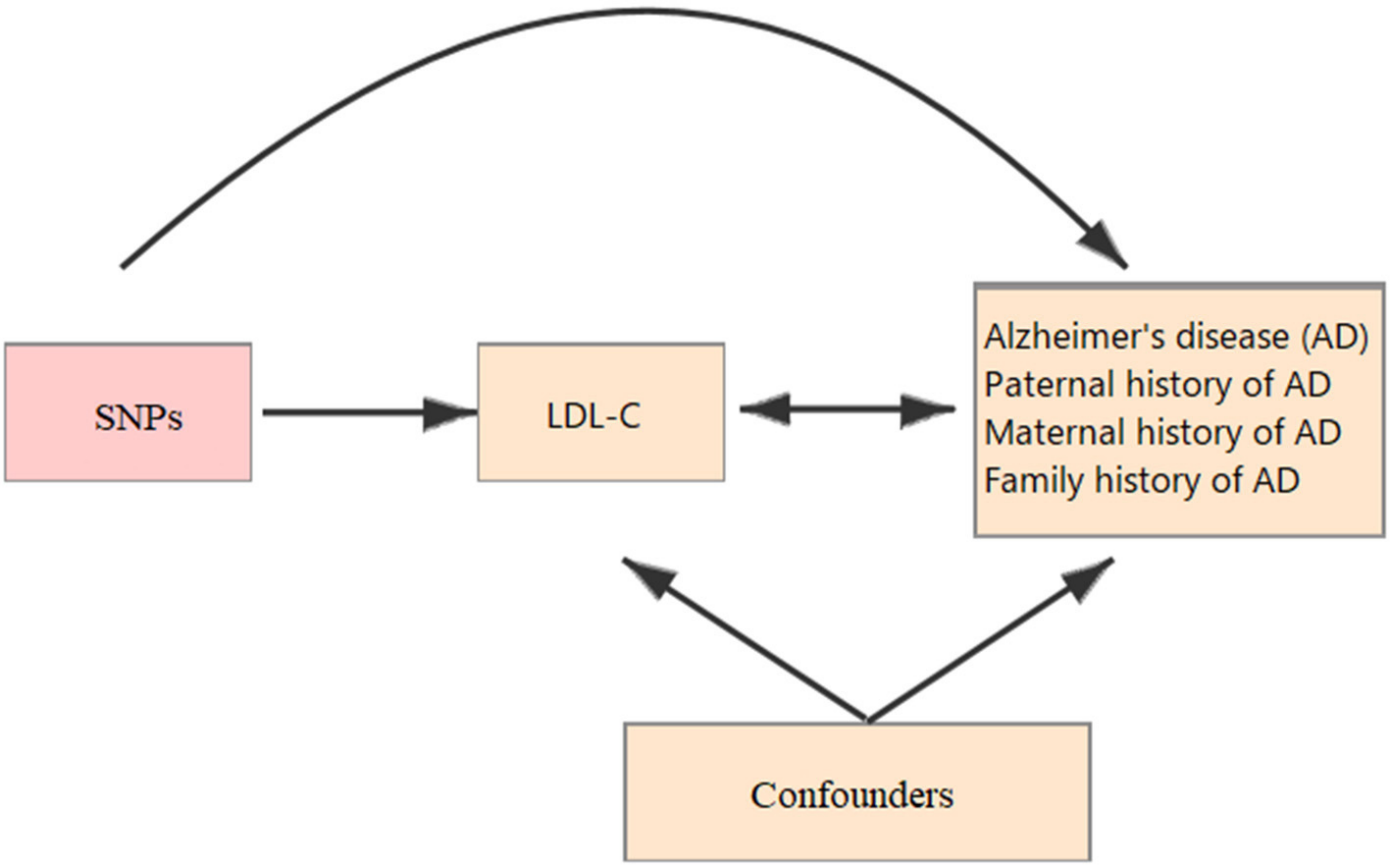
Schematic representation of a Mendelian randomization (MR) analysis. We selected SNPs which associated with low-density lipoprotein cholesterol (LDL-C) and the corresponding effect for these single nucleotide polymorphisms (SNPs) was estimated based on the risk of Alzheimer's disease (AD) obtained from a large cohort of European population.

All the summary data in this study was obtained from published studies. Therefore, the institutional review committees have approved their study in their respective studies, and no further sanction was required. In the present study, two-sample MR (13, 14) was used to assess the causal effect of LDL-C on the risk of AD (Figure 1).

### Data Sources

Summary-level genetic data for LDL-C were acquired from results of the UK Biobank GWAS, which is consisted of 7,221 phenotypes and is available on the Pan UKBB website at https://pan.ukbb.broadinstitute.org/. 144 single nucleotide polymorphisms (SNPs) significantly related to LDL-C were selected as instrumental variables (Supplementary Table 1) based on results from the primary meta-analysis of 343,621 individuals of European ancestry with a genome-wide significant level (*P* < 5 × 10^−8^).

Corresponding data for paternal, maternal, and family history of AD were obtained from the NHGRI-EBI Catalog of human genome-wide association studies (GWAS, https://www.ebi.ac.uk/gwas/). And corresponding data for individual AD was obtained from the MR-Base platform (http://www.mrbase.org) as an alternative because no related SNPs were available from the NHGRI-EBI Catalog of human GWAS.

### Statistical Analysis

As described previously (15), the recently developed method of two-sample MR analyses was used to assess the causal association between LDL-C and AD because no specific individual-level GWAS data was available. To decrease the influence of SNP's heterogeneity on the present study, we used inverse-variance weighted (IVW) meta-analysis in the principal analyses for LDL-C with a random-effects model to combine the instrumental variable ratio estimates across the 144 LDL-C associated SNPs (16). As a first sensitivity analysis, we used the weighted median approach to provide another valid estimate based on a hypothesis that more than 50% of the information come from SNPs which are valid instrumental variables (17). Most importantly, we must ensure that it is our risk factor, but not any other causal pathway, playing an important part in our association between instrumental variables and targeted outcome (also called pleiotropy), which is a fundamental assumption in an MR analysis. Therefore, we used the MR-Egger method to estimate the directional pleiotropy (18) in the second sensitivity analysis. As we all know, IVW, weighted median, and MR-Egger methods are designed to evaluate the effects based on different models of horizontal pleiotropy. The consistency in three different methods can help us judge the reliability of our results (19, 20). Two-tailed *P* < 0.05 was used in the present statistical tests. All statistical analysis was finished based on the R version 4.0.3 (2020-10-10) (The R Foundation for Statistical Computing, Vienna, Austria) and the MR software packages (21, 22).

## Results

### Genetic Instrumental Variables for LDL-C

The baseline characteristics of included studies are shown in Table 1. In brief, the MR study was based on the European population and published between 2018 and 2019. As shown in Supplementary Table 1, we presented all genetic instruments associated with LDL-C on the genome-wide significant level (*P* < 5 × 10^−8^). A total of 144 SNPs were included, and 8 SNPs (rs11591147, rs1229984, rs12740374, rs12916, rs1532085, rs6475606, rs76428106, rs780094) have been reported to be associated with non-AD related diseases, such as coronary artery disease (CAD), MI, and diabetes.

**Table 1:** Baseline characteristics of LDL-C and Alzheimer's disease.

**Trait**	**Year**	**Author**	**Population**	**Sample size**	***n*** **SNP**	***n*** **case**	***n*** **control**
LDL-C	2018	Neale lab	European	–	13586016	–	–
Alzheimer's disease	2019	Kunkle BW	European	63926	10528610	21982	41944
Paternal history of Alzheimer's disease	2018	Marioni RE	European	260279	7776415	14338	245941
Maternal history of Alzheimer's disease	2018	Marioni RE	European	288676	7776415	27696	260980
Family history of Alzheimer's disease	2018	Marioni RE	European	314278	7746640	–	–

*LDL-C, low-density lipoprotein cholesterol; SNP, single nucleotide polymorphism*.

### MR Analysis for the Causal Association

**Figure 2:**
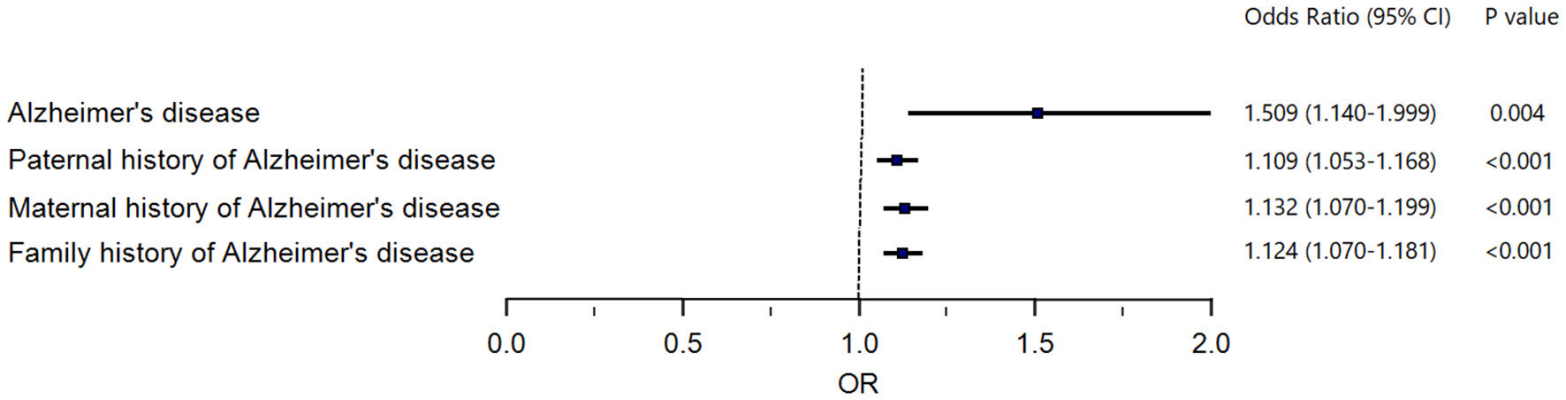
Forest plot to visualize causal effect of LDL-C on the risk of Alzheimer's disease. LDL-C, low-density lipoprotein cholesterol.

We used IVW, MR-Egger, and weighted median regression to estimate causal associations between genetically predicted LDL-C and individual, paternal, maternal, and family history of AD (Figure 2). Genetically predicted LDL-C was significantly positively associated with individual AD [Odds ratio (OR) = 1.509, 95% confidence interval (CI) = 1.140–1.999; *P* = 4.0 × 10^−3^], paternal history of AD [OR = 1.109, 95% CI = 1.053–1.168; *P* = 9.5 × 10^−5^], maternal history of AD [OR = 1.132, 95% CI = 1.070–1.199; *P* = 2.0 × 10^−5^] and family history of AD [OR = 1.124, 95% CI = 1.070–1.181; *P* = 3.7 × 10^−6^] (Figure 2). The OR estimates of the weighted median and MR-Egger analysis (Table 2) were similar to the results of the IVW method but of low precision.

**Table 2:** Associations between genetically predicted LDL-C and Alzheimer's disease in sensitivity analyses using the weighted median and MR-Egger methods.

	**Weighted median**		**MR-egger**	
**Outcome**	**OR (95% CI)**	***P*** **-value**	**OR (95% CI)**	***P*** **-value**
Alzheimer's disease	1.050 (0.903–1.222)	0.527	2.168 (1.398–3.362)	0.001
Paternal history of Alzheimer's disease	1.090 (1.013–1.174)	0.021	1.151 (1.076–1.232)	<0.001
Maternal history of Alzheimer's disease	1.172 (1.106–1.241)	<0.001	1.193 (1.108–1.285)	<0.001
Family history of Alzheimer's disease	1.142 (1.089–1.197)	<0.001	1.178 (1.105–1.256)	<0.001

*LDL-C, low-density lipoprotein cholesterol; CI, confidence interval; OR odds ratio*.

### Analysis of Horizontal Pleiotropy

**Figure 3:**
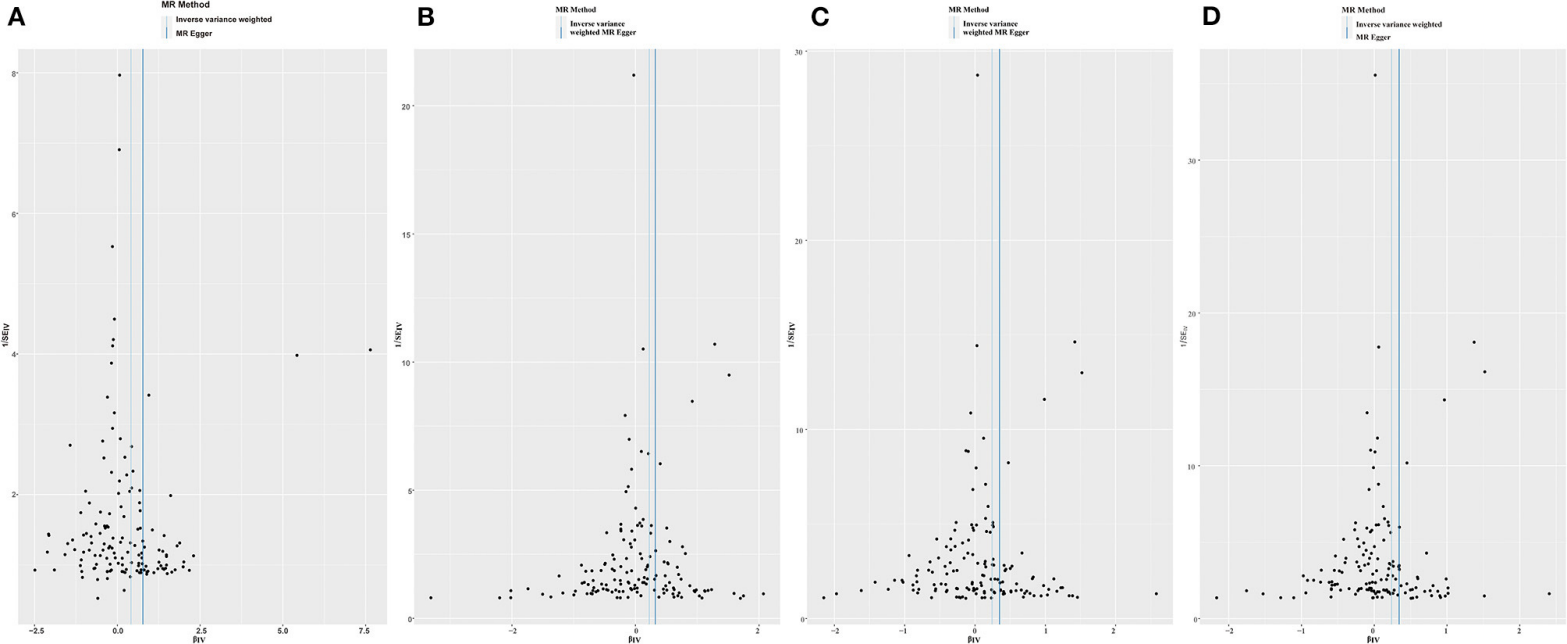
Funnel plots to visualize overall heterogeneity of Mendelian randomization (MR) estimates for the effect of LDL-C on the risk of individual **(A)**, paternal **(B)**, maternal **(C)**, and family **(D)** history of Alzheimer's disease.

Funnel plots may be helpful to detect the individual Wald ratios for each SNP plotted against their precision, where we can see directional horizontal pleiotropy if the asymmetry exists. In the present study, the funnel plots were symmetric so that no horizontal pleiotropy was observed (Figure 3).

### Effects of Individual Genetic Instruments in Relation to Outcomes

Leave-one-out analysis was performed to detect every SNP's influence on our overall causal estimate. In the present study, there was no substantial difference of estimated causal effect observed in the repeated MR analysis when the individual SNP was systematically removed in individual (Supplementary Figure 1), paternal (Supplementary Figure 2), maternal (Supplementary Figure 3), and family (Supplementary Figure 4) history of AD. Therefore, our estimated effects in the present study cannot be explained by any single genetic instrument.

## Discussion

To date, the results of available studies do not allow us to draw a solid conclusion on the causal association between LDL-C level and AD risk. In the present study, by performing the two-sample MR analysis, we identified the causal association between higher LDL-C level and increased AD risk. Compared with previous observational studies prone to confounding and reverse causation, the MR approach is free of reverse causations and confounding factors. Therefore, our MR analysis results may provide direct evidence of the causal role of LDL-C in the development of both individual and familial AD (including paternal, maternal, and family history of AD).

In accordance with our results, a longitudinal study based on elderly Chinese people also revealed that higher LDL-C level was associated with faster global cognitive decline (23). In a nationwide population-based study of 178,586 Korean older adults (age ≥65 years), the researchers pointed out that LDL-C exhibited a positive association with AD risk (24). A meta-analysis including 26 studies, which yielded 2,266 AD patients and 4,767 non-dementia controls, also revealed that the level of LDL-C was higher in AD patients than that of non-dementia controls, implying that serum LDL-C was likely to be a risk factor for AD (25). However, the limitation is that the association between LDL-C level and neurocognitive function in older populations is difficult to interpret as aging is associated with both higher LDL-C and lower cognition (26).

Using multivariable MR analysis, Zhang et al. pointed out that LDL-C was associated with increasing AD risk (OR = 1.193, 95% CI: 1.097–1.296, *P* = 3.564E-05) (27). However, in another two-sample MR, no significant association was observed between LDL-C and AD (*b* = 0.024, *P* = 0.093) (28). Although the F-statistics for all of the analyses were >10 in the two-sample MR, weak instrument bias is in the direction of the null. Moreover, the samples size for outcomes is small, which may result in the MR analysis potentially underpowered. In our two-sample MR, besides exploring the casual association between LDL-C level and individual AD risk, the casual associations between LDL-C level and paternal, maternal, and family history of AD risk were also investigated. Risk estimates from these analyses were all in the same direction, suggesting that a solid causal association was observed between high level of LDL-C and increased risk of AD.

However, two randomized trials examining the impact of lipid-lowering drugs on dementia or cognitive decline yielded disappointing results. The Prospective Study of Pravastatin in the Elderly at Risk (PROSPER) trial recruiting 5,804 subjects aged 70–82 years showed no difference in global cognitive function between patients receiving pravastatin vs. those receiving placebo after four years of follow-up (29). Likewise, there was no significant difference in the incidence of dementia or cognitive performance between patients using simvastatin vs. placebo after five years of follow-up in the Heart Protection Study, where 20,536 UK adults (40–80 years) with coronary disease, other occlusive arterial diseases, or diabetes were included (30). However, dementia or cognitive decline were only regarded as secondary outcomes in both the two trials, and follow-up was relatively short. In a large population-based sample (*n* = 9,294) of older community-dwelling persons with up to 13 years of follow-up, higher LDL-C concentration was associated with an increased risk of AD, and more importantly, the cumulative incidence curves of dementia seem to start separating mostly after five years between patients with high vs. low LDL-C concentrations. This suggests that a longer-term reduction of LDL-C concentration may be required to observe a difference. Moreover, studies have indicated that LDL-C may negatively affect AD only when the increase in LDL-C lasts for a long enough duration (31). Second, the mean age at AD diagnosis is around 85 years (32), and that the pathological processes leading to AD start many years before clinical diagnosis (33), therefore, the age at which LDL-C concentration is measured is critical when exploring the association between LDL-C level and AD risk. Studies reporting a significant association between high LDL-C level and AD risk are mostly studies where LDL-C concentration was measured in midlife (34, 35). In contrast, in studies where lipid was measured in later life, no association (36, 37) or even an inverse relationship with AD risk (38) were observed. Meta-analysis of 26 studies also revealed that in AD patients aged 60–70 years, LDL-C level was higher than that of controls. In contrast, no significant association was observed in patients aged more than 70 years (25). Third, large sample studies revealed a positive association of LDL-C with AD risk, yet no correlation was observed in small sample studies in a meta-analysis (25), implying that sample size may be another confounding factor influencing the association between LDL-C level and AD risk.

The mechanisms of high LDL-C level and AD risk are yet to be fully understood, as the LDL-C level in the brain is independent of peripheral tissues due to the blood-brain barrier. In practice, LDL-C cannot flow directly from the bloodstream into the brain. However, a population-based autopsy study revealed a marked accumulation of 27-hydroxycholesterol (27-OHC) in the brains of AD patients (39). The neurotoxic 27-OHC is an extracerebral metabolite of cholesterol that can crosse the blood-brain barrier. Evidence from matched case-control study and APP/PS1 transgenic mice research revealed that increased flux of 27-OHC to the brain could enhance the accumulation and deposition of Aβ, ultimately accelerating cognitive deficits in AD (40). Second, LDL-C might speed up the metabolism of Aβ, the formation of cortical plaques and tangles, and the creation of neurotoxicity fibrils and neuritis in the human brain, ultimately exacerbating the progression of cognitive impairment related to AD (41). Feeding rabbits a high-cholesterol diet also doubled the number of Aβ plaques, and Aβ degradation was less efficient in a high cholesterol environment (42). Third, high LDL-C level may result in increased permeability of the blood-brain barrier via inflammatory mechanisms. This leads to a consequent and unrestricted leakage of serum cholesterol, inflammatory cytokines, and other amyloidogenic factors into the brain, initiating the process called “neuroinflammation” (43). Neuroinflammation is one of the hallmarks of neurodegenerative diseases, and inhibiting the inflammasome in a mouse model has been proved to improve the clearance of Aβ from the brain (44).

In our two-sample MR analysis, a total of 144 SNPs were included, and 8 SNPs (rs11591147, rs1229984, rs12740374, rs12916, rs1532085, rs6475606, rs76428106, rs780094) have been reported to be associated with non-AD related diseases. Notably, in a study involving 4,251 European patients with CAD and 4,443 controls, the authors pointed out that the rs6475606 (located in the CDKN2B-AS1 gene) was associated with CAD (45). Moreover, Musunuru et al. (46) provided functional evidence that rs12740374 (located in the CELSR2 gene) may alter the risk for MI in humans. It has been well-established that CAD and MI are inflammatory diseases in which immune mechanisms interact with metabolic risk factors (such as LDL-C) to initiate, propagate, and activate lesions in the arterial tree (47, 48). Most importantly, Thackeray et al. (49) revealed that after coronary artery ligation or sham surgery, infarct mice exhibited elevated myocardial mitochondrial translocator protein signal (a marker of activated macrophages and microglia) at one week vs. sham arms. In parallel, brain mitochondrial translocator protein signal was elevated at one week in infarct mice vs. sham arms. This finding highlights a tight interaction between heart and brain inflammation after cardiac injury. The systemic inflammatory response to acute MI may serve as the primer for the subsequent reoccurrence of heart failure, which results in impaired cerebral blood flow and elevated proinflammatory cytokines (50). It has been reported that augmented cerebral tumor necrosis factor-α existed in mice with congestive heart failure, which may lead to cognitive impairment (51). Therefore, it is likely that LDL-C-associated SNPs, such as rs6475606 and rs12740374, also play a significant role in AD patients by the mechanism of inflammation, which is in accordant with the aforementioned “neuroinflammation” mechanism in AD development. In addition, the anti-aging gene Sirtuin 1 can regulate neuron proliferation in various populations and is linked to cardiovascular disease with effects on inflammation, energy, cognition, glucose/cholesterol levels, amyloidosis, and neurogenesis (52, 53). Neurons in the brain with Sirtuin 1 repression may undergo early programmed cell death with altered astrocyte neuron interactions, which may lead to accelerated brain aging (54). Therefore, it seems that Sirtuin 1 may play a significant role in the level of LDL-C and the development of AD. Further researches are required to illustrate the relationship between Sirtuin 1 and AD risk.

Our analysis suggested that elevated level of LDL-C had a causal association with higher risk of AD. In turn, lower level of LDL-C may reduce the risk of AD, as validated by a large-scale MR study including 111,194 individuals (11). The 2016 European guidelines on cardiovascular disease prevention in clinical practice recommend an LDL-C level <1.8 mmol/L in patients at high cardiovascular risk, and <2.6 mmol/L in patients at high risk (55). Previous analysis showed that LDL-C level above 121 mg/dl was positively associated with AD, whereas no association was observed when LDL-C level dropped to 103.9–121 mg/dl (25). Decreasing LDL-C to a very low level should also be of concern, as cholesterol is regarded as the most plentiful type of lipid in the central nervous system, accounting for nearly 25% of the total amount of cholesterol in the body (56). Additionally, cholesterol exerts an essential and vital impact on plasma membrane regionalization, myelin sheath formation, signal transduction, and synaptic formation and maintenance (57). Therefore, an extremely low cholesterol level is potentially detrimental to neurocognitive function. The Framingham Heart Study demonstrated that normal cognitive performance required a certain cholesterol level to maintain (58). Therefore, it is likely that the reduction of LDL-C to different levels is associated with either cognitive impairment or improvement, and striking a balance of LDL-C is of great significance in maintaining cognitive function (59).

Because LDL-C level can be modified by several factors, including diet, exercise, medications, educational level, and lifestyles such as smoking and eating habits, therefore, measures can be applied earlier to prevent worsening cognitive decline potentially when blood lipid level is abnormal. In the future, lowering the LDL-C level is expected to be an important choice in retarding or reversing the condition of cognitive decline in AD. Meanwhile, more randomized trials or cohort researches are necessarily needed to validate the role of LDL-C in the pathogenesis of AD.

### Limitations

The results of this MR study should be interpreted in conjunction with knowledge of its limitations in general. A potential limitation is pleiotropy. That is, a genetic variant is associated with more than one phenotype. However, in our study, the MR-Egger approach was carried out to assess the potential pleiotropic effects among the selected genetic instrumental variables (18). Moreover, weighted median estimator and maximum likelihood method were performed as sensitivity analyses to provide more robust MR estimates (15). In addition, the MR analysis was based on the European population. Whether the results can be extrapolated to other ethnicities need further investigation.

## Conclusions

In conclusion, using the two-sample MR analysis, we identified the causal association between the high level of LDL-C and increased risk of AD. By decreasing LDL-C to a reasonable level, the risk of developing AD may be reduced. We hope that AD can be prevented or delayed by monitoring and modifying the concentration of LDL-C.

## Data Availability Statement

The raw data supporting the conclusions of this article will be made available by the authors, without undue reservation.

## Author Contributions

Y-JY and Y-MY: conception and design and revising for important intellectual content. M-JH and J-ST: analysis and interpretation of data and drafting of the manuscript. final approval of the manuscript submitted was made by J-ST, M-JH, Y-MY, and Y-JY. All authors contributed to the article and approved the submitted version.

## Funding

This work was supported by the National Key Research and Development Program of China (2017YFC1700503), Capital's Funds for Health Improvement and Research (2018-2-4031), and Capital's Funds for Research and Application of Clinical Diagnosis and Treatment Technology (Z191100006619121).

## Conflict of Interest

The authors declare that the research was conducted in the absence of any commercial or financial relationships that could be construed as a potential conflict of interest.

## Publisher's Note

All claims expressed in this article are solely those of the authors and do not necessarily represent those of their affiliated organizations, or those of the publisher, the editors and the reviewers. Any product that may be evaluated in this article, or claim that may be made by its manufacturer, is not guaranteed or endorsed by the publisher.
