# Supplementary figures and images for: Genetic Predisposition to Low-Density Lipoprotein Cholesterol May Increase Risks of Both Individual and Familial Alzheimer's Disease
Source: Front Med (Lausanne). 2022 Jan 11;8:798334. doi: 10.3389/fmed.2021.798334 (PMC8787049; doi:10.3389/fmed.2021.798334)

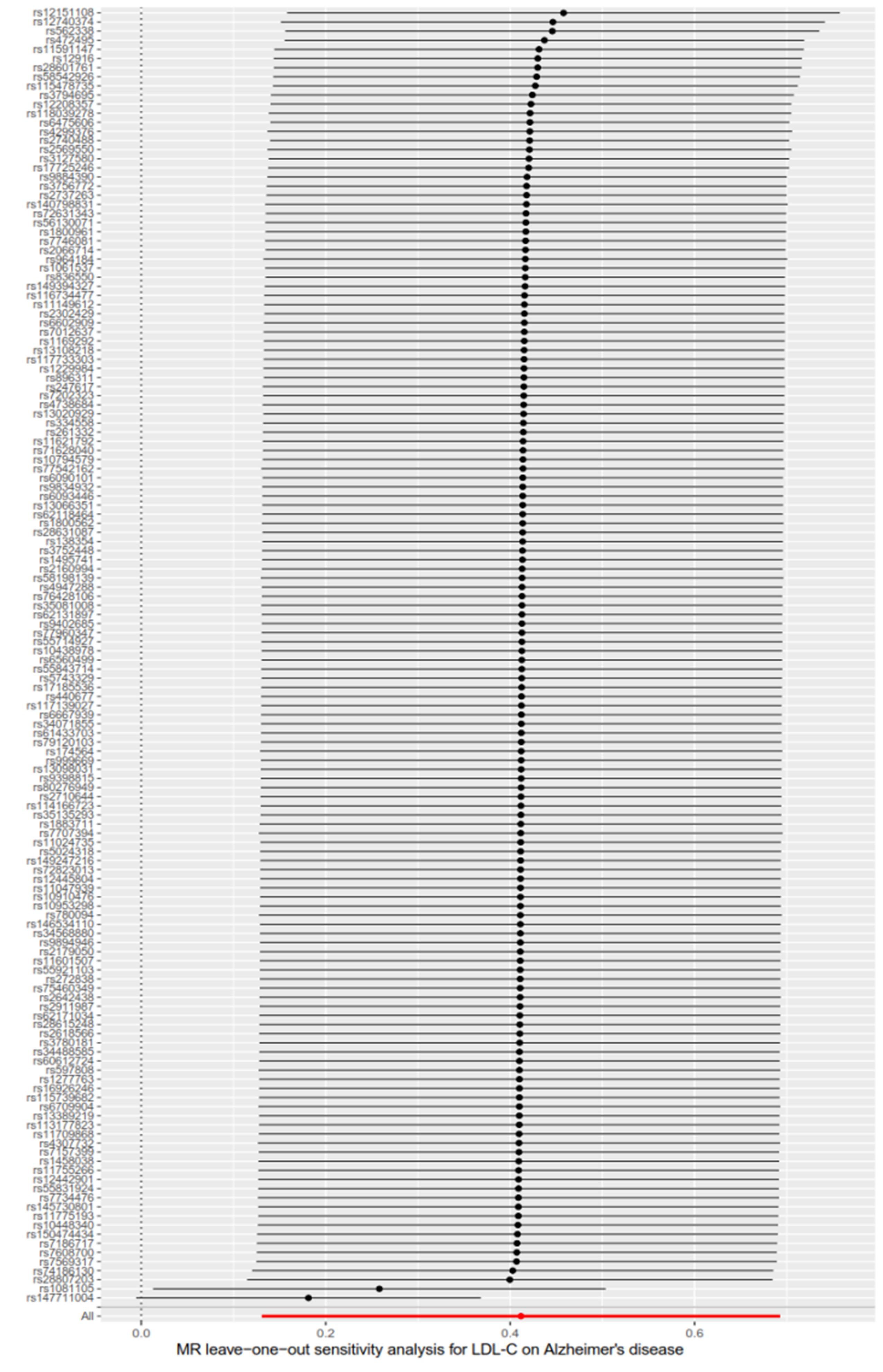

Supplement: Supplementary file 1 [file Image_1.TIF]

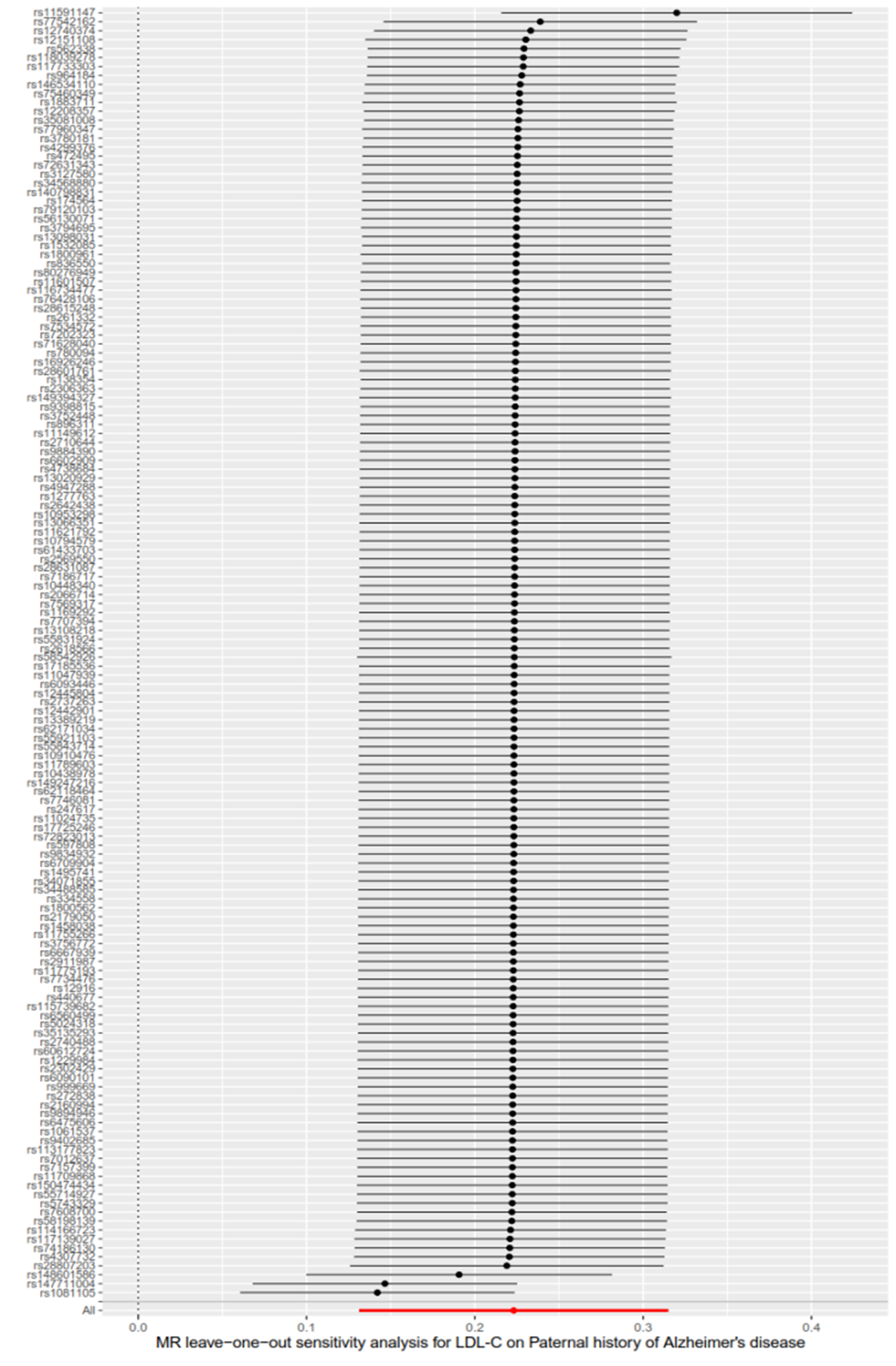

Supplement: Supplementary file 2 [file Image_2.TIF]

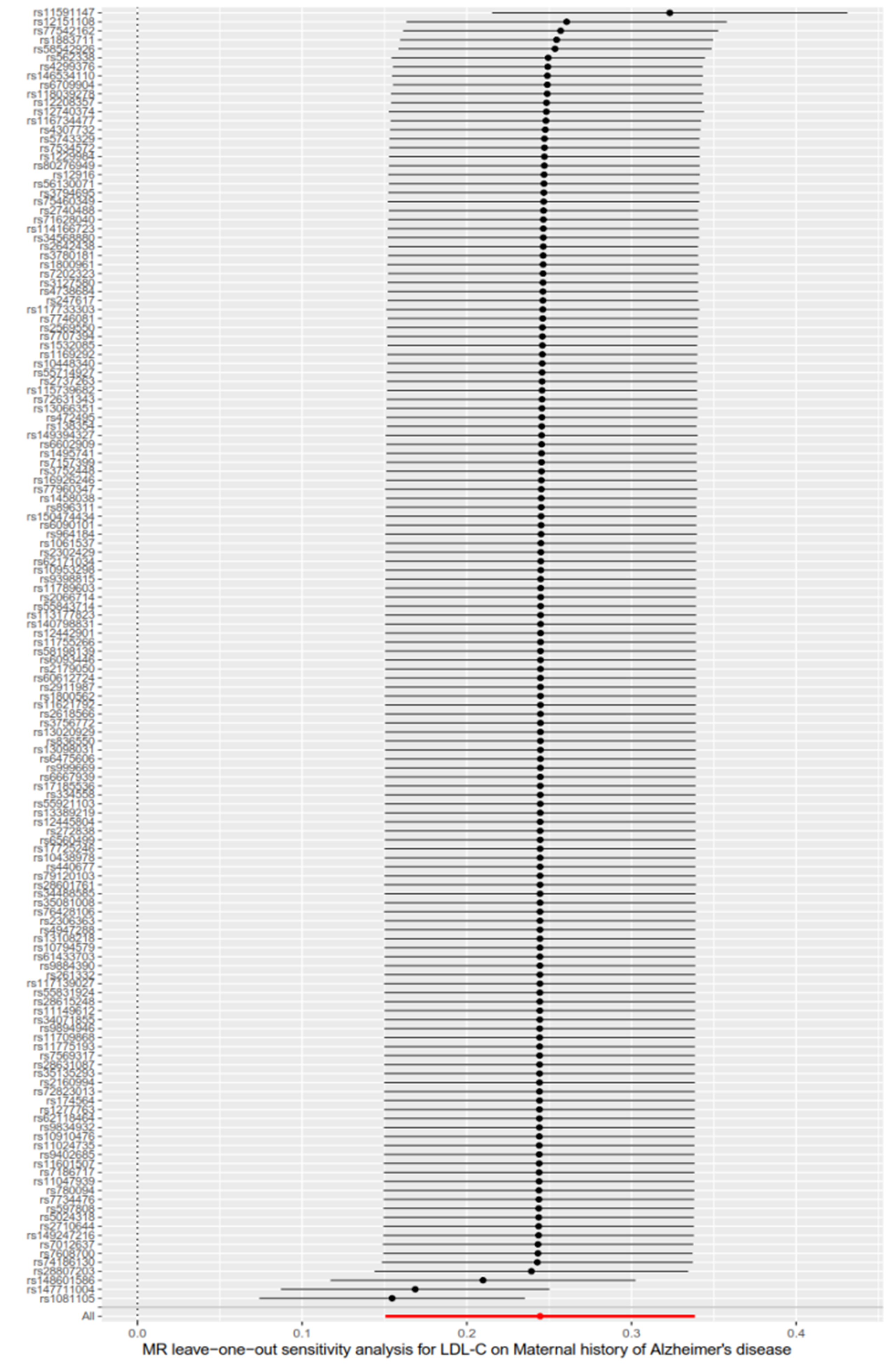

Supplement: Supplementary file 3 [file Image_3.TIF]

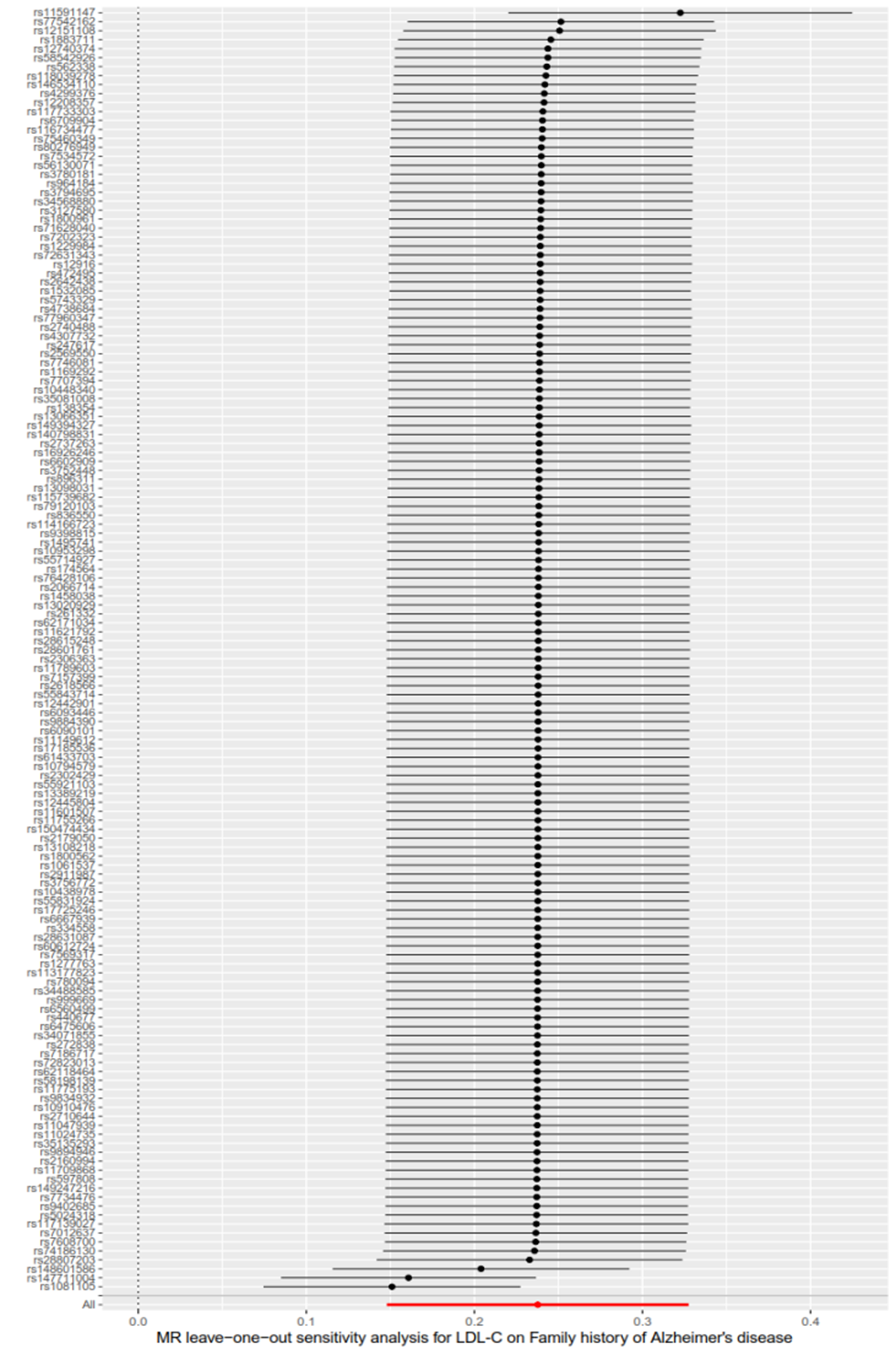

Supplement: Supplementary file 4 [file Image_4.TIF]
