# Supplementary material for: Genetic Predisposition to Low-Density Lipoprotein Cholesterol May Increase Risks of Both Individual and Familial Alzheimer's Disease
Source: Front Med (Lausanne). 2022 Jan 11;8:798334. doi: 10.3389/fmed.2021.798334 (PMC8787049; doi:10.3389/fmed.2021.798334)
Supplement: Supplementary file 5 [file Data_Sheet_1.DOCX]

**Supplementary Table 1. List of genetic instruments for LDL-C and Log Odds Ratios of Alzheimer’s disease risk by each instrumental SNPs (GWAS significance with P<5×10^−8^ and linkage disequilibrium threshold with R^2^<0.005)**

| No. | SNP | Gene | Chr. | EA | OA | LDL-C β (SE) | AD β (SE) | MAD β (SE) | PAD β (SE) | FAD β (SE) |
| --- | --- | --- | --- | --- | --- | --- | --- | --- | --- | --- |
| 1 | rs10438978 | LOC105372112 | 18 | C | T | 0.0175(0.0031) | -0.0038(0.0192) | 0.0048(0.0125) | 0.0005(0.0171) | 0.0033(0.0101) |
| 2 | rs10448340 | - | 9 | G | T | -0.016(0.0025) | -0.0322(0.0154) | 0.0316(0.0123) | 0.0112(0.0168) | 0.0245(0.0099) |
| 3 | rs1061537 | - | 6 | A | G | 0.0185(0.0024) | -0.0238(0.0152) | -0.0073(0.0124) | 0.0217(0.0170) | 0.0028(0.0100) |
| 4 | rs10794579 | - | 10 | C | T | 0.0163(0.0024) | -0.0084(0.0144) | 0.0068(0.0124) | -0.0128(0.0170) | 0.0003(0.0100) |
| 5 | rs1081105 | APOE | 19 | C | A | 0.1736(0.0072) | 0.9420(0.0436) | 0.2465(0.0119) | 0.2229(0.0162) | 0.2383(0.0096) |
| 6 | rs10910476 | - | 1 | T | C | 0.0139(0.0024) | 0.0100(0.0150) | 0.0203(0.0124) | -0.0017(0.0171) | 0.0127(0.0101) |
| 7 | rs10953298 | - | 7 | T | C | -0.0194(0.0028) | -0.0126(0.0170) | 0.0048(0.0123) | 0.0101(0.0169) | 0.0067(0.0099) |
| 8 | rs11024735 | SPTY2D1 | 11 | T | C | 0.0152(0.0027) | 0.0090(0.0166) | 0.0204(0.0126) | 0.0013(0.0172) | 0.0137(0.0102) |
| 9 | rs11047939 | - | 12 | A | G | 0.0176(0.0028) | 0.0114(0.0172) | 0.0205(0.0122) | -0.0035(0.0168) | 0.0122(0.0099) |
| 10 | rs11149612 | LOC105371371 | 16 | T | C | -0.0181(0.0024) | 0.0184(0.0153) | -0.0092(0.0124) | 0.0176(0.0170) | 0.0001(0.0100) |
| 11 | rs113177823 | DNAJC13 | 3 | A | G | -0.037(0.0053) | -0.0608(0.0412) | -0.0054(0.0122) | -0.0172(0.0168) | -0.0095(0.0099) |
| 12 | rs114166723 | SARS1 | 1 | A | G | -0.0498(0.0081) | -0.0195(0.0600) | 0.0046(0.0120) | -0.0277(0.0166) | -0.0065(0.0097) |
| 13 | rs115739682 | PDGFD | 11 | A | T | -0.019(0.0030) | -0.0239(0.0183) | 0.0234(0.0123) | -0.0119(0.0169) | 0.0112(0.0100) |
| 14 | rs11591147 | PCSK9 | 1 | T | G | -0.3485(0.0089) | 0.0304(0.0775) | -0.0125(0.0121) | 0.0097(0.0164) | -0.0047(0.0098) |
| 15 | rs11601507 | TRIM5 | 11 | A | C | 0.0333(0.0046) | 0.0247(0.0303) | 0.0167(0.0126) | -0.0125(0.0173) | 0.0066(0.0102) |
| 16 | rs11621792 | NYNRIN | 14 | T | C | 0.0183(0.0024) | -0.0079(0.0151) | -0.0004(0.0124) | -0.0111(0.0170) | -0.0041(0.0100) |
| 17 | rs116734477 | ITGA1/PELO | 5 | T | C | -0.0512(0.0059) | 0.0399(0.0390) | 0.0176(0.0121) | 0.0006(0.0167) | 0.0117(0.0098) |
| 18 | rs1169292 | HNF1A | 12 | T | C | 0.0241(0.0026) | -0.0093(0.0155) | -0.0199(0.0124) | -0.0029(0.0170) | -0.0140(0.0100) |
| 19 | rs11709868 | LOC100507389 | 3 | T | G | -0.0164(0.0026) | -0.0243(0.0164) | -0.0116(0.0123) | -0.0261(0.0169) | -0.0166(0.0099) |
| 20 | rs117139027 | KIF13B | 8 | A | G | -0.0598(0.0090) | 0.0353(0.1141) | -0.0156(0.0123) | -0.0303(0.0170) | -0.0207(0.0100) |
| 21 | rs11755266 | LOC112267955 | 6 | T | C | -0.0278(0.0040) | -0.0412(0.0248) | -0.0030(0.0121) | -0.0093(0.0166) | -0.0052(0.0098) |
| 22 | rs117733303 | LPAL2 | 6 | G | A | 0.0879(0.0087) | -0.0320(0.0577) | 0.0132(0.0124) | -0.0103(0.0171) | 0.0051(0.0100) |
| 23 | rs11775193 | - | 8 | T | C | -0.0149(0.0025) | -0.0323(0.0161) | -0.0125(0.0123) | -0.0102(0.0169) | -0.0117(0.0099) |
| 24 | rs11789603 | ABCA1 | 9 | T | C | 0.0238(0.0038) | - | -0.0009(0.0125) | 0.0028(0.0170) | 0.0004(0.0101) |
| 25 | rs118039278 | LPA | 6 | A | G | 0.0819(0.0043) | -0.0333(0.0325) | -0.0025(0.0120) | -0.0125(0.0166) | -0.0059(0.0097) |
| 26 | rs12151108 | - | 19 | A | G | -0.1777(0.0036) | -0.0124(0.0223) | -0.0050(0.0123) | -0.0222(0.0169) | -0.0109(0.0100) |
| 27 | rs12208357 | SLC22A1 | 6 | T | C | 0.0569(0.0046) | -0.0546(0.0278) | -0.0163(0.0122) | -0.0133(0.0167) | -0.0153(0.0098) |
| 28 | rs1229984 | ADH1B | 4 | C | T | 0.0545(0.0080) | -0.0171(0.0351) | -0.0187(0.0150) | 0.0179(0.0207) | -0.0061(0.0122) |
| 29 | rs12442901 | CSK | 15 | G | A | 0.0191(0.0027) | 0.0262(0.0160) | -0.0016(0.0125) | -0.0012(0.0171) | -0.0015(0.0101) |
| 30 | rs12445804 | LITAF | 16 | A | G | 0.0261(0.0045) | 0.0186(0.0294) | 0.0060(0.0126) | 0.0014(0.0173) | 0.0044(0.0102) |
| 31 | rs12740374 | CELSR2 | 1 | T | G | -0.1181(0.0028) | -0.0070(0.0171) | -0.0145(0.0124) | 0.0120(0.0169) | -0.0052(0.0100) |
| 32 | rs1277763 | CACNB2 | 10 | C | T | 0.0160(0.0029) | 0.0226(0.0171) | 0.0165(0.0129) | -0.0160(0.0176) | 0.0052(0.0104) |
| 33 | rs12916 | HMGCR | 5 | C | T | 0.0614(0.0024) | -0.0074(0.0146) | -0.0029(0.0124) | 0.0158(0.0170) | 0.0036(0.0100) |
| 34 | rs13020929 | - | 2 | A | G | 0.0142(0.0024) | -0.0158(0.0143) | -0.0025(0.0124) | -0.0194(0.0170) | -0.0084(0.0100) |
| 35 | rs13066351 | PXK | 3 | T | C | -0.0313(0.0043) | 0.0072(0.0248) | 0.0080(0.0123) | 0.0020(0.0169) | 0.0059(0.0100) |
| 36 | rs13098031 | - | 3 | T | G | -0.0194(0.0027) | -0.0061(0.0164) | -0.0005(0.0123) | 0.0337(0.0168) | 0.0114(0.0099) |
| 37 | rs13108218 | HGFAC | 4 | G | A | -0.0187(0.0024) | 0.0159(0.0155) | -0.0068(0.0124) | 0.0060(0.0170) | -0.0024(0.0100) |
| 38 | rs13389219 | COBLL1 | 2 | T | C | -0.0138(0.0024) | -0.0201(0.0145) | -0.0019(0.0125) | 0.0041(0.0171) | 0.0002(0.0101) |
| 39 | rs138354 | XPNPEP3 | 22 | C | T | -0.0136(0.0024) | 0.0092(0.0143) | 0.0293(0.0124) | 0.0300(0.0170) | 0.0295(0.0100) |
| 40 | rs140798831 | - | 2 | C | T | -0.0354(0.0025) | 0.0062(0.0153) | -0.0049(0.0123) | 0.0176(0.0168) | 0.0029(0.0099) |
| 41 | rs1458038 | - | 4 | T | C | -0.0193(0.0026) | -0.0243(0.0159) | 0.0108(0.0123) | -0.0087(0.0169) | 0.0040(0.0099) |
| 42 | rs146534110 | SLC22A1 | 6 | T | G | 0.0627(0.0103) | 0.0508(0.0676) | -0.0168(0.0123) | -0.0147(0.0171) | -0.0161(0.0100) |
| 43 | rs147711004 | - | 19 | A | G | 0.1486(0.0064) | 1.1354(0.0366) | 0.2260(0.0114) | 0.2252(0.0156) | 0.2257(0.0092) |
| 44 | rs148601586 | - | 19 | G | C | 0.1345(0.0104) | - | 0.1328(0.0116) | 0.1238(0.0159) | 0.1296(0.0094) |
| 45 | rs149247216 | - | 2 | C | A | 0.0263(0.0047) | 0.0176(0.0296) | 0.0218(0.0120) | 0.0038(0.0165) | 0.0156(0.0097) |
| 46 | rs149394327 | - | 17 | C | G | 0.0654(0.0070) | -0.0519(0.0466) | 0.0098(0.0124) | 0.0082(0.0169) | 0.0092(0.0100) |
| 47 | rs1495741 | - | 8 | A | G | -0.0188(0.0028) | 0.0060(0.0172) | 0.0165(0.0125) | -0.0058(0.0171) | 0.0087(0.0101) |
| 48 | rs150474434 | INSIG2/LOC107985940 | 2 | A | G | -0.0357(0.0039) | -0.0504(0.0252) | -0.0014(0.0122) | -0.0189(0.0168) | -0.0074(0.0099) |
| 49 | rs1532085 | - | 15 | G | A | -0.0184(0.0024) | - | 0.0298(0.0124) | 0.0371(0.0171) | 0.0323(0.0101) |
| 50 | rs16926246 | HK1 | 10 | T | C | -0.0221(0.0035) | -0.0291(0.0224) | 0.0092(0.0123) | 0.0169(0.0168) | 0.0119(0.0099) |
| 51 | rs17185536 | - | 6 | T | C | -0.0176(0.0027) | -0.0005(0.0172) | -0.0023(0.0123) | 0.0046(0.0169) | 0.0001(0.0099) |
| 52 | rs174564 | FADS2 | 11 | G | A | -0.0313(0.0025) | -0.0117(0.0153) | -0.0134(0.0124) | 0.0221(0.0169) | -0.0010(0.0100) |
| 53 | rs17725246 | NPC1L1 | 7 | C | T | 0.0353(0.0030) | -0.0298(0.0188) | 0.0087(0.0124) | 0.0071(0.0170) | 0.0081(0.0100) |
| 54 | rs1800562 | HFE/HFE-AS1 | 6 | A | G | -0.0552(0.0044) | -0.0062(0.0302) | -0.0119(0.0121) | -0.0136(0.0166) | -0.0125(0.0098) |
| 55 | rs1800961 | HNF4A | 20 | T | C | -0.0600(0.0067) | 0.0560(0.0415) | -0.0013(0.0121) | -0.0020(0.0166) | -0.0016(0.0098) |
| 56 | rs1883711 | - | 20 | C | G | 0.1080(0.0069) | 0.0465(0.0516) | -0.0139(0.0122) | 0.0103(0.0166) | -0.0054(0.0098) |
| 57 | rs2066714 | ABCA1 | 9 | C | T | 0.0238(0.0035) | -0.0377(0.0208) | -0.0010(0.0131) | -0.0057(0.0179) | -0.0026(0.0105) |
| 58 | rs2160994 | LIMA1 | 12 | C | T | 0.0179(0.0025) | -0.0017(0.0153) | 0.0132(0.0123) | 0.0201(0.0169) | 0.0156(0.0100) |
| 59 | rs2179050 | HMGXB4 | 22 | G | A | -0.0142(0.0026) | -0.0127(0.0158) | 0.0037(0.0125) | -0.0089(0.0172) | -0.0006(0.0101) |
| 60 | rs2302429 | POR | 7 | A | G | 0.0175(0.0030) | - | -0.0071(0.0123) | 0.0169(0.0169) | 0.0012(0.0100) |
| 61 | rs2306363 | SIPA1 | 11 | T | G | 0.0159(0.0029) | -0.0333(0.0189) | 0.0064(0.0123) | -0.0239(0.0169) | -0.0041(0.0100) |
| 62 | rs247617 | - | 16 | A | C | -0.0340(0.0025) | -0.0022(0.0155) | 0.0145(0.0123) | -0.0065(0.0169) | 0.0072(0.0100) |
| 63 | rs2569550 | LDLR | 19 | C | T | 0.0427(0.0024) | -0.0062(0.0145) | -0.0043(0.0123) | 0.0034(0.0169) | -0.0017(0.0100) |
| 64 | rs261332 | LIPC/LIPC-AS1 | 15 | G | A | -0.0232(0.0029) | 0.0092(0.0179) | -0.0083(0.0123) | 0.0201(0.0169) | 0.0015(0.0100) |
| 65 | rs2618566 | - | 20 | T | G | -0.0234(0.0025) | -0.0163(0.0154) | -0.0019(0.0124) | 0.0025(0.0170) | -0.0004(0.0100) |
| 66 | rs2642438 | MTARC1 | 1 | G | A | 0.0249(0.0026) | 0.0154(0.0165) | -0.0285(0.0123) | -0.0061(0.0169) | -0.0207(0.0099) |
| 67 | rs2710644 | EHBP1 | 2 | C | A | 0.0202(0.0026) | 0.0080(0.0154) | 0.0252(0.0124) | -0.0136(0.0169) | 0.0116(0.0100) |
| 68 | rs272838 | SLC22A4 | 5 | T | C | -0.0189(0.0032) | -0.0164(0.0193) | -0.0045(0.0125) | -0.0186(0.0171) | -0.0094(0.0101) |
| 69 | rs2737263 | TRPS1 | 8 | T | G | -0.0217(0.0026) | 0.0293(0.0160) | 0.0191(0.0122) | 0.0002(0.0168) | 0.0126(0.0099) |
| 70 | rs2740488 | ABCA1 | 9 | C | A | -0.0238(0.0027) | 0.0493(0.0168) | 0.0339(0.0124) | -0.0132(0.0171) | 0.0176(0.0100) |
| 71 | rs28601761 | - | 8 | G | C | -0.0609(0.0024) | 0.0087(0.0148) | -0.0153(0.0124) | -0.0062(0.0169) | -0.0121(0.0100) |
| 72 | rs28615248 | RP1 | 8 | C | T | 0.0238(0.0030) | 0.0195(0.0190) | 0.0094(0.0123) | -0.0197(0.0170) | -0.0006(0.0100) |
| 73 | rs28631087 | LOC107985366 | 1 | C | T | -0.0167(0.0029) | 0.0176(0.0203) | -0.0131(0.0126) | 0.0120(0.0172) | -0.0043(0.0101) |
| 74 | rs28807203 | CEACAM19/CEACAM16-AS1 | 19 | C | A | -0.1222(0.0055) | -0.1163(0.0358) | -0.0580(0.0148) | -0.0490(0.0203) | -0.0549(0.0120) |
| 75 | rs2911987 | AGPAT5/MCPH1-AS1 | 8 | G | A | 0.0139(0.0025) | 0.0153(0.0156) | -0.0031(0.0123) | 0.0104(0.0169) | 0.0015(0.0100) |
| 76 | rs3127580 | - | 6 | T | C | 0.0359(0.0032) | -0.0396(0.0206) | -0.0127(0.0122) | -0.0176(0.0167) | -0.0144(0.0098) |
| 77 | rs334558 | GSK3B/LOC107986119 | 3 | G | A | 0.0171(0.0025) | -0.0115(0.0151) | 0.0023(0.0127) | 0.0079(0.0174) | 0.0042(0.0103) |
| 78 | rs34071855 | CASZ1 | 1 | G | C | -0.0140(0.0025) | -0.0020(0.0155) | -0.0097(0.0124) | -0.0053(0.017) | -0.0082(0.0100) |
| 79 | rs34488585 | SEC1P | 19 | T | C | -0.0258(0.0043) | -0.0389(0.0295) | -0.0073(0.0124) | -0.0080(0.0169) | -0.0076(0.0100) |
| 80 | rs34568880 | ATP6V1G2/NFKBIL1/ ATP6V1G2-DDX39B | 6 | T | C | 0.0601(0.0104) | 0.0453(0.0592) | -0.0012(0.0129) | -0.0035(0.0176) | -0.002(0.0104) |
| 81 | rs35081008 | ZNF329 | 19 | T | C | -0.0349(0.0033) | -0.0070(0.0207) | -0.0094(0.0124) | 0.0305(0.0168) | 0.0046(0.0100) |
| 82 | rs35135293 | - | 2 | T | C | -0.0148(0.0024) | -0.0063(0.0143) | -0.0139(0.0124) | -0.0153(0.0169) | -0.0144(0.0100) |
| 83 | rs3752448 | N4BP2L1 | 13 | A | T | -0.0225(0.0031) | 0.0036(0.0182) | 0.0108(0.0123) | 0.0123(0.0169) | 0.0114(0.0100) |
| 84 | rs3756772 | FRK | 6 | T | C | 0.0188(0.0024) | -0.0281(0.0144) | -0.0001(0.0124) | 0.0095(0.017) | 0.0032(0.0100) |
| 85 | rs3780181 | VLDLR | 9 | G | A | -0.0285(0.0047) | -0.0349(0.0302) | 0.0238(0.0126) | 0.0353(0.0173) | 0.0278(0.0102) |
| 86 | rs3794695 | HPR/TXNL4B | 16 | T | C | 0.0500(0.0030) | -0.0221(0.0181) | -0.0088(0.0125) | -0.0042(0.0172) | -0.0072(0.0101) |
| 87 | rs4299376 | ABCG8/LOC102725159 | 2 | T | G | -0.0519(0.0025) | 0.0047(0.0164) | 0.0282(0.0123) | 0.0099(0.0169) | 0.0218(0.0100) |
| 88 | rs4307732 | ST3GAL4 | 11 | A | G | 0.0475(0.0038) | 0.0322(0.0231) | -0.0193(0.0125) | 0.0362(0.0170) | 0.0001(0.0101) |
| 89 | rs440677 | KANK2 | 19 | A | G | -0.0169(0.0024) | -0.0025(0.0154) | -0.0047(0.0124) | -0.0119(0.0170) | -0.0072(0.0100) |
| 90 | rs472495 | PCSK9 | 1 | T | G | 0.0422(0.0025) | -0.0601(0.0156) | -0.0013(0.0125) | -0.0155(0.0170) | -0.0063(0.0101) |
| 91 | rs4738684 | - | 8 | G | A | -0.0303(0.0025) | -0.0011(0.0150) | 0.0183(0.0123) | 0.0042(0.0169) | 0.0134(0.0100) |
| 92 | rs4947288 | FLOT1 | 6 | G | A | 0.0184(0.0028) | -0.0040(0.0182) | 0.0067(0.0124) | -0.0126(0.0170) | - |
| 93 | rs5024318 | GPAM | 10 | A | T | 0.0200(0.0027) | -0.0106(0.0157) | -0.0245(0.0123) | -0.0123(0.0169) | -0.0203(0.0100) |
| 94 | rs55714927 | ASGR1 | 17 | T | C | -0.0288(0.0030) | -0.0028(0.0218) | 0.0115(0.0122) | -0.0201(0.0169) | 0.0006(0.0099) |
| 95 | rs55831924 | PLEC | 8 | T | C | 0.0175(0.0025) | 0.0259(0.0153) | 0.0090(0.0124) | -0.0066(0.0170) | 0.0035(0.0100) |
| 96 | rs55843714 | CYP26C1 | 10 | T | C | 0.0203(0.0024) | 0.0031(0.0147) | -0.0023(0.0126) | 0.0006(0.0172) | -0.0013(0.0102) |
| 97 | rs55921103 | MITF | 3 | T | G | 0.0137(0.0025) | 0.0127(0.0151) | 0.0014(0.0125) | -0.0034(0.0171) | -0.0003(0.0101) |
| 98 | rs56130071 | D-H11 | 7 | C | G | 0.0313(0.0029) | -0.0160(0.0179) | -0.0256(0.0123) | -0.0175(0.0168) | -0.0228(0.0099) |
| 99 | rs562338 | - | 2 | G | A | 0.1017(0.0031) | -0.0151(0.0184) | 0.0017(0.0128) | -0.0064(0.0175) | -0.0011(0.0103) |
| 100 | rs5743329 | NOD1 | 7 | C | A | -0.0359(0.0065) | 0.0108(0.0446) | 0.0229(0.0120) | -0.0187(0.0166) | 0.0086(0.0097) |
| 101 | rs58198139 | - | 5 | T | C | 0.0340(0.0024) | 0.0096(0.0149) | 0.0052(0.0124) | 0.0230(0.0170) | 0.0114(0.0100) |
| 102 | rs58542926 | TM6SF2 | 19 | T | C | -0.1080(0.0045) | 0.0191(0.0279) | 0.0103(0.0122) | -0.0227(0.0168) | -0.0011(0.0099) |
| 103 | rs597808 | ATXN2 | 12 | G | A | 0.0264(0.0024) | 0.0182(0.0149) | 0.0215(0.0125) | 0.0051(0.0171) | 0.0158(0.0101) |
| 104 | rs60612724 | MYH16 | 7 | G | A | 0.0361(0.0062) | 0.0495(0.0381) | 0.0046(0.0159) | 0.0174(0.0218) | 0.0091(0.0129) |
| 105 | rs6090101 | - | 20 | A | G | 0.0182(0.0030) | -0.0115(0.0181) | -0.0103(0.0124) | 0.0173(0.0170) | -0.0007(0.0100) |
| 106 | rs6093446 | PLCG1 | 20 | A | G | 0.0200(0.0026) | -0.0052(0.0161) | -0.0004(0.0125) | -0.0015(0.0172) | -0.0008(0.0101) |
| 107 | rs61433703 | LOC107985440 | 20 | A | G | 0.0179(0.0032) | 0.0026(0.0198) | 0.0073(0.0126) | -0.0114(0.0173) | 0.0008(0.0102) |
| 108 | rs62118464 | PPP1R37 | 19 | A | G | 0.0316(0.0037) | -0.0223(0.0333) | 0.0139(0.0121) | 0.0053(0.0166) | 0.0109(0.0098) |
| 109 | rs62171034 | ABCB11 | 2 | T | A | -0.0197(0.0024) | -0.0151(0.0147) | 0.0052(0.0124) | 0.0003(0.0170) | 0.0035(0.0100) |
| 110 | rs6475606 | CDKN2B-AS1 | 9 | T | C | -0.0217(0.0024) | 0.0451(0.0151) | -0.002(0.0125) | -0.0191(0.0171) | -0.008(0.0101) |
| 111 | rs6560499 | PCSK5 | 9 | A | G | -0.0134(0.0024) | 0.0024(0.0145) | -0.0031(0.0124) | -0.0145(0.0169) | -0.0071(0.0100) |
| 112 | rs6602909 | GAS6 | 13 | C | T | 0.0225(0.0025) | -0.0117(0.0155) | -0.013(0.0126) | -0.0106(0.0172) | -0.0122(0.0102) |
| 113 | rs6667939 | LINC01221 | 1 | T | C | 0.0162(0.0026) | 0.0025(0.0162) | 0.0003(0.0124) | 0.0098(0.0170) | 0.0036(0.0100) |
| 114 | rs6709904 | ABCG8 | 2 | G | A | -0.0418(0.0038) | -0.0280(0.0222) | 0.0392(0.0126) | -0.0098(0.0173) | 0.0223(0.0102) |
| 115 | rs7012637 | - | 8 | A | G | 0.0259(0.0024) | -0.0061(0.015) | 0.0291(0.0124) | 0.0192(0.0170) | 0.0256(0.0100) |
| 116 | rs7157399 | SYNJ2BP/SYNJ2BP-COX16 | 14 | C | T | 0.0190(0.0034) | 0.0331(0.0204) | -0.0152(0.0123) | 0.0234(0.0169) | -0.0019(0.0100) |
| 117 | rs71628040 | - | 5 | C | T | -0.0306(0.0051) | 0.0329(0.0338) | 0.0202(0.0120) | 0.0104(0.0164) | 0.0168(0.0097) |
| 118 | rs7186717 | LOC105371334 | 16 | T | C | 0.0191(0.0024) | 0.0344(0.0150) | 0.0200(0.0124) | -0.0089(0.0170) | 0.0100(0.0100) |
| 119 | rs7202323 | - | 16 | G | T | -0.0255(0.0028) | 0.0071(0.0166) | 0.0265(0.0126) | 0.0175(0.0173) | 0.0234(0.0102) |
| 120 | rs72631343 | ABCA10 | 17 | G | C | -0.0315(0.0035) | 0.0321(0.0221) | 0.0096(0.0125) | 0.0253(0.0171) | 0.0151(0.0101) |
| 121 | rs72823013 | LOC105378493 | 10 | A | G | -0.0223(0.0035) | -0.0143(0.0226) | -0.0132(0.0122) | -0.0039(0.0167) | -0.0100(0.0098) |
| 122 | rs74186130 | HLA-DQB1 | 6 | G | C | 0.0433(0.0034) | 0.0697(0.0218) | 0.0290(0.0125) | 0.0350(0.0171) | 0.0311(0.0101) |
| 123 | rs7534572 | DOCK7 | 1 | G | C | 0.0399(0.0025) | - | -0.0207(0.0124) | -0.0058(0.0171) | -0.0155(0.0101) |
| 124 | rs75460349 | ZDHHC18 | 1 | C | A | 0.0580(0.0079) | 0.0427(0.0497) | -0.0017(0.0121) | -0.0137(0.0167) | -0.0059(0.0098) |
| 125 | rs7569317 | FAM117B | 2 | C | T | 0.0186(0.0024) | 0.0351(0.0142) | 0.0123(0.0124) | -0.0083(0.0170) | 0.0051(0.0100) |
| 126 | rs7608700 | ACMSD | 2 | A | C | 0.0166(0.0026) | 0.0382(0.0147) | 0.0428(0.0127) | 0.0344(0.0174) | 0.0399(0.0102) |
| 127 | rs76428106 | FLT3 | 13 | C | T | -0.0626(0.0107) | 0.0373(0.0791) | -0.0158(0.0123) | -0.0044(0.0168) | -0.0118(0.0099) |
| 128 | rs7707394 | ANKRD31 | 5 | A | G | 0.0402(0.0025) | 0.0173(0.0150) | -0.0063(0.0126) | 0.0041(0.0173) | -0.0026(0.0102) |
| 129 | rs7734476 | CSNK1G3 | 5 | A | G | 0.0216(0.0024) | 0.0228(0.0144) | 0.0210(0.0124) | 0.0106(0.0170) | 0.0174(0.0100) |
| 130 | rs7746081 | - | 6 | A | G | -0.0242(0.0026) | 0.0157(0.0153) | 0.0248(0.0126) | -0.0034(0.0173) | 0.0150(0.0102) |
| 131 | rs77542162 | ABCA6 | 17 | G | A | 0.1306(0.0079) | 0.0298(0.0516) | -0.0080(0.0120) | -0.0218(0.0165) | -0.0128(0.0097) |
| 132 | rs77960347 | LIPG | 18 | G | A | 0.0711(0.0102) | -0.0044(0.0647) | 0.0133(0.0120) | 0.0003(0.0165) | 0.0088(0.0097) |
| 133 | rs780094 | GCKR | 2 | C | T | -0.0343(0.0024) | -0.0162(0.0147) | -0.0181(0.0124) | 0.0081(0.0170) | -0.0090(0.0100) |
| 134 | rs79120103 | - | 18 | G | A | -0.0379(0.0068) | -0.0071(0.0421) | -0.0098(0.0121) | 0.0131(0.0165) | -0.0018(0.0097) |
| 135 | rs80276949 | SGMS1 | 10 | A | G | 0.0456(0.0079) | 0.0094(0.0716) | -0.0129(0.0120) | -0.003(0.0164) | -0.0095(0.0097) |
| 136 | rs836550 | RAC1 | 7 | G | A | 0.0137(0.0024) | -0.0341(0.0148) | -0.0028(0.0124) | -0.0454(0.0171) | -0.0176(0.0100) |
| 137 | rs896311 | - | 7 | A | G | 0.0168(0.0026) | -0.0183(0.0157) | -0.0137(0.0124) | -0.0198(0.0170) | -0.0158(0.0100) |
| 138 | rs9398815 | - | 6 | C | T | -0.0137(0.0024) | -0.0037(0.0146) | 0.0084(0.0124) | 0.0276(0.0170) | 0.0151(0.0100) |
| 139 | rs9402685 | - | 6 | C | T | -0.0148(0.0027) | 0.0045(0.0164) | -0.0205(0.0123) | -0.0259(0.0169) | -0.0224(0.0100) |
| 140 | rs964184 | ZPR1 | 11 | C | G | -0.0578(0.0035) | -0.0053(0.0207) | -0.0095(0.0126) | 0.0269(0.0173) | 0.0031(0.0102) |
| 141 | rs9834932 | CMTM6 | 3 | G | A | -0.0351(0.0041) | 0.0046(0.0254) | -0.0145(0.0125) | -0.0072(0.0171) | -0.0120(0.0101) |
| 142 | rs9884390 | - | 4 | C | T | 0.0252(0.0028) | -0.0532(0.0213) | 0.0081(0.0122) | -0.0085(0.0167) | 0.0024(0.0099) |
| 143 | rs9894946 | - | 17 | G | A | -0.0182(0.0033) | -0.0162(0.0210) | -0.0106(0.0124) | -0.0204(0.0169) | -0.0140(0.0100) |
| 144 | rs999669 | - | 2 | C | T | 0.0133(0.0024) | 0.0031(0.0149) | -0.0017(0.0124) | 0.0226(0.0170) | 0.0067(0.0100) |

Chr. indicates chromosome; EA, effect allele; FAD: familial Alzheimer’s disease; LDL-C, low-density lipoprotein cholesterol; MAD, maternal Alzheimer’s disease; OA, other allele; PAD, paternal Alzheimer’s disease; SNP, single nucleotide polymorphism
