# Supplementary material for: Genetic Predisposition to Low-Density Lipoprotein Cholesterol May Increase Risks of Both Individual and Familial Alzheimer's Disease
Source: Front Med (Lausanne). 2022 Jan 11;8:798334. doi: 10.3389/fmed.2021.798334 (PMC8787049; doi:10.3389/fmed.2021.798334)
Supplement: Supplementary file 6 [file Table_1.DOCX]

**Supplementary Figure 1** Leave-one-out plot to visualize causal effect of LDL on the risk of Individual Alzheimer's disease when leaving one SNP out.

**Supplementary Figure 2** Leave-one-out plot to visualize causal effect of LDL on the risk of paternal history of Alzheimer's disease when leaving one SNP out

**Supplementary Figure 3** Leave-one-out plot to visualize causal effect of LDL on the risk of maternal history of Alzheimer's disease when leaving one SNP out

**Supplementary Figure 4** Leave-one-out plot to visualize causal effect of LDL on the risk of family history of Alzheimer's disease when leaving one SNP out
